# Integrated insular phenotype (IIP) versus Berger–Sanai and Yasargil classifications: comparative prognostic value in surgery of insular gliomas

**DOI:** 10.1007/s00701-025-06744-9

**Published:** 2025-12-11

**Authors:** Valentyn Kliuchka

**Affiliations:** Department of Innovative Neurosurgical Technologies, The State Institution Romodanov Neurosurgery Institute National Academy of Medical Sciences of Ukraine, 32 Platona Mayborody St, Kyiv, 04050 Ukraine

**Keywords:** Insular glioma, Brain neoplasms, Prognostic factors, Extent of resection, Seizure control, Neurological deficit

## Abstract

**Background:**

Surgery of insular gliomas remains one of the most demanding areas in neuro-oncology. Traditional classifications (Berger–Sanai, Yasargil) have limited prognostic value. We evaluated the Integrated Insular Phenotype (IIP), an ordinal system reflecting tumor complexity and its associations with surgical outcomes.

**Methods:**

We retrospectively analyzed 167 patients with histologically confirmed insular gliomas. For each case, tumor topography was classified according to Berger–Sanai, Yasargil, and IIP systems. Three binary outcomes were assessed: extent of resection (EOR), seizure control, and persistent neurological deficit at day 90. Logistic regression models were applied to evaluate associations, and performance was assessed using odds ratios (OR), AUC, AIC, and LR χ^2^.

**Results:**

Increasing IIP complexity was associated with reduced likelihood of total/subtotal resection (OR = 3.83; *p* < 0.001), poorer seizure control (OR = 2.90; *p* < 0.001), and higher risk of persistent deficits (OR = 2.83; *p* = 0.004). IIP showed lower AIC and higher LR χ^2^ values compared with Berger–Sanai and Yasargil, indicating superior prognostic performance. While Berger–Sanai yielded high point estimates, confidence intervals were wide, and Yasargil produced consistent but less discriminative results.

**Conclusions:**

In this retrospective cohort, the Integrated Insular Phenotype (IIP) showed superior prognostic performance compared with the Berger–Sanai and Yasargil classifications. IIP more accurately reflected topographic complexity and may assist in balancing oncological radicality with functional safety. Further prospective multicenter validation is warranted.

## Introduction

Surgery of insular gliomas remains one of the most challenging areas in modern neuro-oncology. The anatomical proximity to critical vascular structures and major white matter pathways entails a high risk of intraoperative complications and postoperative neurological deficits [4, 11, 19]. At the same time, this tumor category is characterized by a high incidence of epileptic seizures, highlighting the dual goals of surgery: achieving maximal safe oncological resection while providing effective seizure control [15, 16].

Over the past decades, several classifications have been proposed to systematize the topographic variants of insular gliomas. The most widely recognized are the Berger–Sanai zonal classification [19] and the Yasargil typology [23]. The former divides the insula into four quadrants, providing only a rough approximation of surgical risk based on localization, but it does not account for multizonal involvement, tumor volume, or extension into neighboring cortical areas. The Yasargil system focuses on pathways of tumor spread into adjacent cortical and subcortical regions; however, it lacks gradation of surgical complexity, does not incorporate functional or vascular barriers, and provides limited prognostic value for postoperative outcomes. Despite their historical importance, both systems therefore fail to fully capture the heterogeneity of insular gliomas, and their associative utility with respect to oncological and functional outcomes remains restricted [1, 13].

Recent meta-analyses and multicenter studies have shown that surgical outcomes in insular gliomas are determined not only by the extent of resection but also by the complexity of anatomical and functional barriers that limit access to the tumor [1, 2, 5, 10, 13]. Moreover, seizure control and the risk of persistent neurological deficits vary substantially depending on tumor spread and surgical strategies employed [3, 7, 9, 18, 20, 21].

In this context, we proposed the Integrated Insular Phenotype (IIP), an ordinal system that classifies tumors along a gradient of complexity—from simple localized variants (L) to complex multizonal and widespread ones (M). Unlike previous classifications, IIP integrates key topographic features and provides more reproducible prognostic associations with surgical outcomes.

The aim of this study was to compare the prognostic performance of IIP with the traditional Berger–Sanai and Yasargil classifications in relation to three major surgical outcomes: extent of resection, seizure control, and persistent neurological deficit at 90 days.

This study focused exclusively on the topographic and prognostic aspects of the Integrated Insular Phenotype (IIP) and did not address biological, surgical-technical, or epileptological parameters, which are analyzed separately.

## Methods

### Patient population

A total of 167 patients with insular gliomas who underwent microsurgical resection at a specialized neurosurgical center were included. The study cohort was collected between 2019 and early 2024, with a minimum clinical and radiological follow-up of 12 months for all included patients (database locked in May 2025). Data were derived from a prospectively maintained institutional database but analyzed retrospectively.

This study was conducted in accordance with the Declaration of Helsinki. Given its retrospective design and use of anonymized clinical data, formal ethics approval was waived according to institutional policy. Written informed consent for data use was obtained from all patients or their legal representatives.

Inclusion criteria were histologically confirmed insular glioma, availability of preoperative neuroimaging, and complete clinical information on seizure history and postoperative neurological status. Patients with incomplete data or those who had undergone repeat interventions were excluded.

Preoperative DTI-tractography was performed in 41 of 167 patients (24.6%) and functional MRI in 36 of 167 (21.6%) to assess the spatial relationship between the tumor and motor or language pathways. Intraoperative motor monitoring (motor evoked potentials and direct cortical/subcortical stimulation) was used in 31 of 167 cases (18.6%), while awake mapping was performed in 11 patients (6.6%). Neuronavigation was employed in 80 of 167 procedures (47.9%). Functional and navigational techniques were applied selectively according to the predicted risk profile, consistent with the principle of maximal safe resection.

Although the present study focuses on the prognostic value of topographic classifications (IIP, Berger–Sanai, and Yasargil), and intraoperative techniques were not included as variables in the comparative statistical analysis, these data are provided to clarify the surgical context.

### Topographic classification

For each case, three classification systems were applied:

- Berger–Sanai (BS): division of the insula into four quadrants (I–IV).
- Yasargil typology: classification of tumor spread (types 3A–5B).
- Integrated Insular Phenotype (IIP): an ordinal measure of surgical complexity with three levels:
  o IIP-L (Local): tumor confined to a single insular zone according to BS, without spread beyond the insular cortex (Yasargil 3A–3B).
  o IIP-H (Hybrid): multizonal involvement within the insula (≥ 2 BS zones) or extension to one adjacent region (e.g., frontoorbital or temporopolar), without medial basal involvement (Yasargil 5A without medial base).
  o IIP-M (Multizonal): tumor involving multiple insular zones and ≥ 2 neighboring cortical regions, or critical areas (medial basal or opercular regions), corresponding to Yasargil 5B with medial base involvement.

### Endpoints

Three binary outcomes were evaluated:

1. Extent of resection (EOR): total/subtotal vs. partial.
2. Seizure control: according to the International League Against Epilepsy (ILAE) classification — grades 1–2 (favorable control) vs. grades 3–5 (unfavorable control).
3. Persistent neurological deficit at day 90 (Deficit90): present vs. absent.

### Statistical analysis

For each classification system, separate logistic regression models were built with the corresponding endpoint.

Model effects were expressed as odds ratios (OR) with 95% confidence intervals (CI) and p-values; area under the receiver operating characteristic curve (*AUC*), a measure of model discrimination; Akaike Information Criterion (*AIC*), a measure of model fit penalized for the number of parameters; and likelihood ratio chi-square (*LR χ*^*2*^), evaluating model informativeness compared with the null model.

The prognostic performance of IIP was compared with the Berger–Sanai and Yasargil classifications based on AUC, AIC, LR χ^2^, and the stability of OR estimates.

All statistical analyses were performed using IBM SPSS Statistics, Version 23.0 (IBM Corp., Armonk, NY, USA). Descriptive statistics were reported as means with standard deviations or medians with interquartile ranges, as appropriate. Associations between categorical variables were assessed using the χ^2^ test or Fisher’s exact test. Continuous variables were compared using the Student’s t-test or Mann–Whitney U test, depending on distribution.

Logistic regression models were applied to evaluate factors associated with extent of resection, seizure control, and persistent neurological deficit at 90 days.

Results are presented as odds ratios (OR) with 95% confidence intervals (CI). Model performance was assessed using the area under the receiver operating characteristic curve (AUC). For visualization, forest plots were used to display odds ratios, and bar charts were used to compare AUC values across classification systems.

A *p*-value < 0.05 was considered statistically significant.

Given the retrospective nature of this study, all analyses were exploratory and aimed to describe associations rather than establish causality or predictive models.

## Results

### Patient distribution

Patient distribution across the three classification systems reflected the heterogeneity of insular gliomas.

- In the Berger–Sanai classification, tumors were located in quadrant I in 58 patients (34.7%), quadrant II in 41 (24.6%), quadrant III in 43 (25.7%), and quadrant IV in 25 (15.0%).
- According to the Yasargil typology, 45 patients (26.9%) were type 3 A, 37 (22.2%) type 3B, 23 (13.8%) type 5 A, and 62 (37.1%) type 5B.
- The Integrated Insular Phenotype (IIP) produced a balanced distribution: 46 patients (27.5%) were classified as IIP-L, 58 (34.7%) as IIP-H, and 63 (37.7%) as IIP-M.

Calibration analysis for all three models showed good concordance between observed and expected outcomes, without systematic over- or underestimation, underscoring the robustness of the IIP-based associations.

### Extent of resection (EOR)

In the model based on the Integrated Insular Phenotype (IIP), higher levels of complexity were significantly associated with a reduced likelihood of achieving total or subtotal resection (OR = 3.83; 95% CI 2.39–6.14; *p* < 0.001). The IIP model showed the highest informativeness (LR χ^2^ = 38.7) and the lowest AIC (196.1), reflecting superior parsimony compared with the Berger–Sanai and Yasargil classifications.

The Yasargil classification showed a similar but less pronounced association (OR = 2.23; 95% CI 1.67–2.97; *p* < 0.001), whereas the Berger–Sanai model revealed strong associations in quadrants III and IV (OR = 8.12 and OR = 4.00, respectively). However, these were accompanied by wide confidence intervals and markedly higher AIC and lower LR χ^2^ values (Table 1).

**Table 1:** Extent of resection (EOR) according to Integrated Insular Phenotype (IIP), Yasargil and Berger–Sanai classifications

Scale	OR [95% CI]	*p*	AUC	AIC	LR χ^2^
IIP (L → H → M)	3.83 [2.39–6.14]	< 0.001	0.749	196.1	38.7
Yasargil (3A → 5B)	2.23 [1.67–2.97]	< 0.001	0.746	200.5	34.3
Berger–Sanai (ref = I)	II: 2.71 [1.15–6.41]; III: 8.12 [3.31–19.92]; IV: 4.00 [1.48–10.79]	< 0.001 (global)	0.71	213.9	24.8

Logistic regression results with odds ratios (OR), 95% confidence intervals (CI), p-values, and model performance metrics (AUC, AIC, LR χ^2^)

### Seizure control

IIP also showed a significant association with postoperative seizure control. Increasing complexity of the phenotype was associated with a higher risk of unfavorable seizure outcomes (ILAE 3–5) (OR = 2.90; 95% CI 1.70–4.94; *p* < 0.001). The IIP model achieved greater informativeness (LR χ^2^ = 18.1) and a lower AIC (140.7) compared with Yasargil (LR χ^2^ = 14.5; AIC = 144.3).

**Fig. 1:**
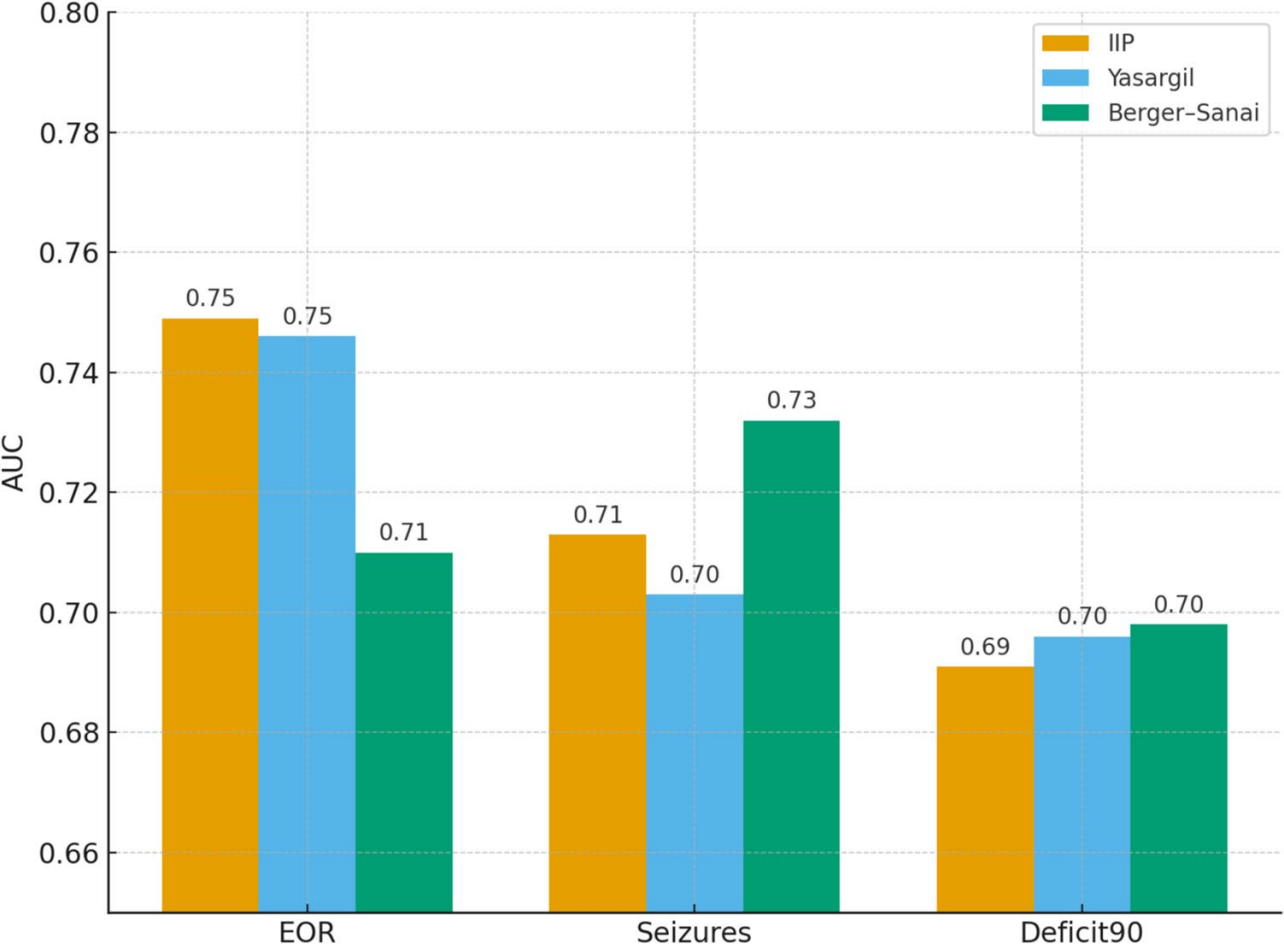
Comparison of discriminative ability (AUC) between IIP, Yasargil and Berger–Sanai models for three outcomes: extent of resection (EOR), seizure control, and persistent neurological deficit (Deficit90)

In this subgroup, the Berger–Sanai classification produced high point estimates (e.g., OR = 12.11 for quadrant III), but these were accompanied by wide confidence intervals and unstable estimates. Despite yielding a slightly higher AUC (0.732 vs. 0.713 for IIP), the overall model performance was inferior (Table 2, Fig. 1).

**Table 2:** Seizure control (ILAE 1–2 vs 3–5) predicted by IIP, Yasargil and Berger–Sanai classifications

Scale	OR [95% CI]	*p*	AUC	AIC	LR χ^2^
IIP (L → H → M)	2.90 [1.70–4.94]	< 0.001	0.713	140.7	18.1
Yasargil (3A → 5B)	1.85 [1.32–2.59]	< 0.001	0.703	144.3	14.5
Berger–Sanai (ref = I)	II: 5.22 [1.59–17.19]; III: 12.11 [3.66–40.12]; IV: 5.92 [1.59–22.11]	< 0.001 (global)	0.732	141.2	21.6

Results of logistic regression with OR, 95% CI, p-values, AUC, AIC, and LR χ^2^

### Persistent neurological deficit at day 90

In the IIP model, higher levels of complexity were significantly associated with an increased risk of persistent neurological deficit (OR = 2.83; 95% CI 1.40–5.73; *p* = 0.004). This model achieved the lowest AIC (123.8) and an LR χ^2^ of 10.3, indicating an optimal balance between accuracy and parsimony.

The Yasargil classification showed a weaker but still significant effect (OR = 1.94; *p* = 0.004), whereas the Berger–Sanai model again produced inflated ORs with very wide confidence intervals (e.g., OR = 18.00 for quadrant IV), limiting its practical value (Table 3).

**Table 3:** Persistent neurological deficit at 90 days (Deficit90) according to IIP, Yasargil and Berger–Sanai classifications

Scale	OR [95% CI]	*p*	AUC	AIC	LR χ^2^
IIP (L → H → M)	2.83 [1.40–5.73]	0.004	0.691	123.8	10.3
Yasargil (3A → 5B)	1.94 [1.24–3.03]	0.004	0.696	124.0	10.2
Berger–Sanai (ref = I)	II: 11.74 [1.38–99.53]; III: 13.03 [1.56–108.66]; IV: 18.00 [2.04–159.19]	< 0.001 (global)	0.698	124.5	13.7

Logistic regression results with OR, 95% CI, p-values, AUC, AIC, and LR χ^2^

### Comparative analysis of models

In the overall comparison, the Integrated Insular Phenotype (IIP) was the only system that consistently demonstrated stable and reproducible prognostic associations across all three endpoints. While the Berger–Sanai classification yielded the highest individual odds ratios (ORs), they were accompanied by wide confidence intervals and high AIC values, indicating potential overfitting and limited generalizability. The Yasargil classification provided more consistent results but showed lower LR χ^2^ values and weaker discriminatory power.

**Fig. 2:**
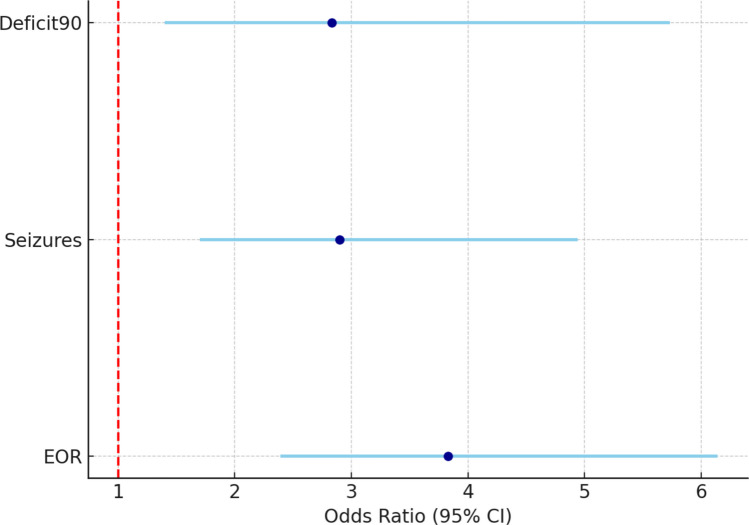
Forest plot of odds ratios for Integrated Insular Phenotype (IIP) across three major outcomes (EOR, seizure control, Deficit90). Higher IIP complexity consistently predicted worse outcomes, with all 95% confidence intervals lying above 1

The bar plot of AUC values (Fig. 1) illustrates that the discriminatory ability of IIP is comparable to Yasargil and slightly lower than Berger–Sanai for seizure control, yet consistently superior in overall model performance and stability. The forest plot for IIP (Fig. 2) confirms that this parameter is associated with all key outcomes (EOR, seizure control, Deficit90), with narrow confidence intervals and odds ratios greater than 1.

## Discussion

In our study, the Integrated Insular Phenotype (IIP) showed stable and reproducible associations with three key surgical outcomes in insular gliomas: extent of resection, seizure control, and the risk of persistent neurological deficit at 90 days. Unlike the traditional Berger–Sanai and Yasargil classifications, IIP provided higher discriminatory ability (AUC), lower AIC values, and greater informativeness according to the LR χ^2^ criterion. Higher IIP levels (M) were associated with less radical resections, lower likelihood of seizure control, and increased risk of persistent deficits, whereas simple phenotypes (L) corresponded to the most favorable outcomes.

This study focused exclusively on the comparative prognostic value of topographic classifications. Biological, surgical-technical, and epileptological parameters were not analyzed in this work.

Comparison with published data supported the clinical relevance of our observations. In the meta-analysis by Alturki et al. (2019), the mean extent of resection for insular gliomas was approximately 80%, seizure freedom was achieved in about 65% of cases, and persistent deficits were observed in 15–20% of patients [1]. Similar findings were reported in modern series by Ng (2021) and Morshed (2020) [12, 13]. In our cohort, simple IIP phenotypes achieved outcomes comparable to these benchmarks, whereas complex phenotypes showed lower resectability and poorer seizure control, reflecting real anatomic and functional limitations.

Anatomical and functional barriers remained the principal determinants of surgical outcomes in insular glioma surgery. Vascular structures, including M2 branches and LSA perforators, as well as the internal capsule and critical language tracts, limited the radicality of resection and defined the risk of irreversible deficits [4, 6, 11, 17, 18, 24]. The traditional Berger–Sanai and Yasargil systems merely described localization or tumor spread direction but did not integrate multizonal involvement or extrainsular extension, which frequently determined surgical complexity [2, 19, 23]. In contrast, IIP incorporated these key topographic features into a single ordinal scale that more accurately reflected the balance between achievable radicality and functional safety.

Our findings had direct clinical implications. The principle of “maximal safe resection” remained a cornerstone of modern neuro-oncology [5, 10]. Excessive pursuit of total resection in complex phenotypes (IIP-M) carried an unacceptably high risk of permanent deficits, consistent with the findings of Southwell et al. [21], who reported persistent neurological morbidity in one-quarter of patients following high-complexity resections [21]. Conversely, in IIP-L phenotypes, total resection was achieved without significant functional compromise. Thus, IIP may help surgeons individualize surgical strategies depending on the expected balance between radicality and functional safety.

Special attention should be given to seizure outcomes. Epileptic manifestations were the predominant symptom in most patients with gliomas [15, 16]. Previous studies showed that surgical efficacy depended on the extent of epileptogenic resection: De Witt Hamer et al. (2013) demonstrated a strong correlation between completeness of resection and seizure reduction [3], while Pallud et al. (2014) reported seizure control in two-thirds of patients after radical procedures [15]. In our cohort, multizonal and widespread forms (IIP-M) were associated with poorer seizure control, likely reflecting diffuse epileptogenic networks that could not be completely eliminated. These results were consistent with Englot et al. (2011), who noted worse outcomes in lesions affecting multiple functional areas [9].

Modern technologies have expanded the potential of surgical treatment. Functional MRI, DTI tractography, and awake mapping increased the extent of resection without proportionally raising the rate of permanent complications [8, 24]. Nevertheless, even with these tools, the primary limiting factor remained tumor topographic complexity. In this context, IIP may serve as a useful risk stratification tool and a guide for determining the need for awake surgery or combined approaches.

Strengths of our study included the use of a prospectively maintained database, standardized endpoints (EOR, seizures, Deficit90), and consistent application of regression models, ensuring high-quality data and minimizing systematic bias.

However, several limitations should be acknowledged: this was a single-center retrospective study, limiting generalizability; IIP requires external validation in independent cohorts; and the sample size was smaller compared with large multicenter meta-analyses [1, 12, 14].

This analysis focused exclusively on topographic predictors. Molecular and histopathological variables were not incorporated, as the study was designed to isolate and test the topographic component of prognostic variability. Correlations between IIP, IDH status, and tumor grade are being analyzed separately within the same institutional cohort.

Epileptological outcomes according to histological and molecular subtypes are also being investigated in a separate analysis, as these factors were beyond the topographic focus of the present study.

Neurocognitive outcomes, which are also important determinants of quality of life and long-term prognosis in glioma patients, were not evaluated in the present analysis [22].

The study was not intended to explore detailed surgical techniques or epilepsy mechanisms.

Taken together, our data suggest that IIP is a simple yet informative prognostic tool that shows better performance than the traditional Berger–Sanai and Yasargil classifications. Its application may contribute to the individualization of surgical strategies, allowing a balance between oncological radicality and functional safety, while improving the assessment of seizure outcomes.

Future studies should focus on external validation of IIP and its integration with modern intraoperative technologies. Another promising avenue is the application of artificial intelligence and machine learning (AI/ML) algorithms for risk stratification, with IIP serving as a central topographic variable. Such approaches may also enable the development of prognostic nomograms, further supporting personalized surgical planning and patient counseling.

These findings should be interpreted within the retrospective and exploratory nature of the study, and further prospective validation is warranted.

## Conclusions

The Integrated Insular Phenotype (IIP) showed superior prognostic performance compared with the traditional Berger–Sanai and Yasargil classifications in association with three key surgical outcomes for insular gliomas: extent of resection, seizure control, and persistent neurological deficit at 90 days.

As an ordinal integrated system, IIP more accurately reflected the topographic complexity of insular tumors by incorporating anatomical–functional features and multizonal extension. This provided greater reproducibility of statistical models and suggested practical value for surgical planning.

Our findings suggested that IIP may serve as a helpful risk stratification tool, supporting individualized decision-making by balancing oncological radicality against functional safety. Further multicenter studies are required to validate its prognostic value and to promote integration with biological markers and modern intraoperative technologies.

These conclusions should be interpreted within the retrospective and exploratory nature of the study.

## Data Availability

The datasets generated and/or analyzed during the current study are not publicly available due to patient privacy and institutional regulations but are available from the corresponding author on reasonable request.
